# Effect of biosurfactant sophorolipids on *Rhizomucor miehei* lipase fermentation by *Aspergillus oryzae*

**DOI:** 10.1186/s40643-021-00433-y

**Published:** 2021-09-03

**Authors:** Qianqian Zhang, Zhiyue Xiong, Lei Sun, Xiwei Tian, Guiwei Tian, Yiming Yang, Xu Li, Yonghong Wang, Ju Chu

**Affiliations:** 1 State Key Laboratory of Bioreactor Engineering, East China University of Science and Technology, 130 Meilong Road, P.O. box 329, Shanghai, 200237 People’s Republic of China; 2 Wilmar Biotechnology R&D Center Co., Ltd, Shanghai, 200137 China

**Keywords:** *Rhizomucor miehei* lipase, *Aspergillus oryzae*, Sophorolipids, Morphology, Amino acid

## Abstract

In this study, the effect of biosurfactant sophorolipids (SLs) on *Rhizomucor miehei* lipase (RML) fermentation by *Aspergillus oryzae* was investigated. With the exogenous addition of 0.3% (w/v) SLs in the initial medium, the RML activity reached 430.0 U/mL, an increase of 25.0% compared to the control group. Subsequently, the physiological metabolic responses of *A. oryzae* to the addition of SLs were further explored. The results showed that though SLs had almost no effect on the RML secretion, it would affect the morphology of the cells. During the late phase of the fermentation, the proportion of middle pellets, which was generally considered as an energetic and stable state for enzyme production was increased with the addition of SLs. Simultaneously, the viscosity of fermentation broth was reduced, which facilitated the increase of oxygen transfer, thereby improving the RML production. Finally, it could be found that the addition of SLs significantly increased the contents of precursor amino acids, especially for those rank first and second of the RML composition, and it could promote the synthesis of RML.

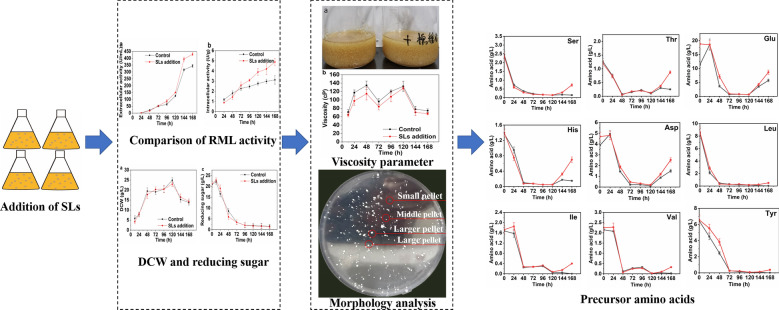

## Introduction

*Rhizomucor miehei* lipase (RML) is an *α*/*β*-type single-chain polypeptide composed of 269 amino acids with a Ser-Asp-His catalytic triad structure (Boel et al. 1988; Huge-Jensen et al. 1989; Turkenburg et al. 1990). Due to its excellent interface activation, water-soluble RML can efficiently catalyze hydrolysis, transesterification, esterification and other reactions in non-aqueous reaction systems, so it has broad applications in oleochemistry, food processing, medicine, detergents and other industries (Sarmah et al. 2018; Torres and Otero 2001). However, the lipase production capacity of wild type *R. miehei* is relatively low, and the composition of the enzyme solution is unstable. Therefore, the RML production cost is pretty high, which greatly limits the industrial applications (Miao et al. 2013; Zhang et al. 2010). Researchers have been focusing on constructing heterologous expression systems for efficient RML production either by *Aspergillus oryzae* (Wang et al. 2009) or *Pichia pastoris* (Han et al. 2009; Liu et al. 2014).

*Aspergillus oryzae*, which is generally recognized as safe, is one of the important cell factories for protein production. However, compared with *P. pastoris* as the expression host of heterologous RML production, which could reach approximate 1200 U/mL (Huang et al. 2014), the expression level in *A. oryzae* seems to be much lower. Wang et al. (2009) successfully expressed RML in *A. oryzae* and obtained the integrated positive transformant *A. oryzae* ONL1, however, the enzyme activity of the fermentation supernatant measured by alkali titration was only 2.5 U/mL after 7 days cultivation.

Surfactants are a class of organic compounds with hydrophilic and lipophilic properties. They have the functions of dispersion, emulsification, defoaming, penetration, solubilization, wetting, and adsorption. There have been many reports using surfactants as fermentation promoters for the production of amino acids (Huchenq et al. 1984), enzymes (Reddy et al. 1999) and new materials (Galindo and Salcedo 1996). Surfactants could improve the oxygen transfer at the gas–liquid interface and the permeability of cell membranes (Zhou 2012). Huchenq et al. (1984) studied the effect of surfactants on the fermentation of glutamate and found that surfactants have an inhibitory effect on the synthesis of fatty acids in the cell membrane, which resulted in changing the composition of the cell membrane and consequently increased the permeability to glutamate. As a kind of biosurfactant, SLs is a secondary metabolite produced by *Candida* sp*.* and has the advantages of low toxicity, biodegradability, high temperature and salt resistances (Li et al. 2020). Xu et al. (2011) mentioned that the addition of SLs could induce the production efficiency of cellulase fermentation. And sophorose played a primary role as an effective inducer of cellulase synthesis.

In this study, the effects of five surfactants on the RML production by *A. oryzae* were investigated, and then the cellular metabolic responses to exogenous SLs addition for increasing RML activity were further explored from the perspectives of the broth rheological characteristics, the cell morphology and the cellular physiological metabolism.

## Materials and methods

### Strain, media and cell cultivations

*Aspergillus oryzae* was kindly provided by Wilmar Biotechnology R&D Center Co., Ltd, Shanghai, China.

The seed medium (g/L) consisted of corn dextrin 20, corn steep powder 10, yeast extract powder 1, polyether defoamer 1, KH_2_PO_4_ 5, MgSO_4_·7H_2_O 0.5, Na_2_HPO_4_·12H_2_O 1. The composition of the fermentation medium (g/L) was as follows: corn dextrin 40, peptone 30, yeast extract powder 4, polyether defoamer 1, KH_2_PO_4_ 1.12, MgSO_4_·7H_2_O 0.5, Na_2_HPO_4_·12H_2_O 1.

*A. oryzae* spore suspension was inoculated into 250-mL shake flasks containing the seed medium. After incubating at 28 °C on a rotary shaker (150 rpm) for 28 h, the seed solution was transferred into 250-mL shake flasks containing fermentation medium with 10% inoculum. The flasks were incubated at 28 °C on a rotary shaker (200 rpm) for 168 h.

### Screening of the surfactants and optimization of the SLs addition amount

According to the addition amount of 0.2% (w/v), five surfactants including Tween-80, acacia, polyvinyl alcohol, Triton X-100 and SLs (lactonic type, Lihan Biotech Co. Ltd, Shanghai, China) were added to the fermentation medium, respectively. Then, the flasks were incubated at 28℃ on a rotary shaker (200 rpm) for 168 h. In detail, the SLs addition amount was set as 0.1%, 0.15%, 0.2%, 0.25%, 0.3%, 0.35% and 0.5%, respectively. Each set of experiments were repeated for three times.

### Extracellular RML enzyme activity assay

RML activity in the diluted fermentation supernatant was determined by the *p*-nitrophenol (*p*-NP) colorimetric method. One unit of RML activity was defined as the amount of enzyme, which liberated 1 μmol of *p*-NP from *p*-nitrophenol palmitate (*p*-NPP) per minute. The *p*-NPP solution and the substrate buffer containing phosphate buffer saline (PBS) were mixed in a ratio of 1:9 (v/v). 2.4 mL of the mixed solution was pipetted into a 5 mL EP tube, and all tubes were preheated at 37 °C for 3 min. Next, 100 μL of the diluted fermentation supernatant was added to the test tube, and 100 μL of the boiled denatured sample solution was added to the control tube as a control. All the tubes were put in a constant temperature water bath oscillator at 37 °C for 15 min. To stop the reaction, 2 mL of 95% ethanol was added immediately. Finally, all the tubes were centrifuged at 4000 rpm for 5 min. The absorbance of the sample and the control group were measured at 410 nm. In addition, *p*-NP standard solutions of different concentrations were prepared and measured by same method. And a *p*-NP standard curve was acquired. RML enzyme activity (U/mL) was calculated according to formula (1):

1 $${\text{Enzyme activity }} = \frac{{\left( {A_{1} - A_{0} } \right) + 0.0015}}{2.4684*15*0.1}*n,$$

where *A*_*1*_ is the absorbance of the sample, *A*_*0*_ is the absorbance of the corresponding control group, *n* is the dilution factor of the fermentation supernatant.

### Intracellular RML enzyme activity assay

Pedersen's method was referred and modified slightly for intracellular RML enzyme activity assay (Pedersen et al. 1999). About 0.07 g of wet mycelia was weighted in a 2-mL EP tube, and 1 mL of 0.2 M pH 8.0 PBS containing 1 mM ethylene diamine tetraacetic acid (EDTA) was added to resuspend the mycelia. Then, 500 μL of grinding beads (0.5 mm in diameter) were added in each EP tube, and all the tubes were put in a freezing grinder. The frequency and time were set as 65 Hz and 4 min, respectively. Finally, all the samples were centrifuged at 12,000 rpm for 2 min. The supernatant was acquired and the enzyme activity was determined by colorimetry.

Specifically, to acquire accurate intracellular activity proportion, the units of intracellular and extracellular enzyme activity had been normalized into units in the whole fermentation broth.

### Dry cell weight (DCW) assay

The biomass of *A. oryzae* was indicated by the DCW. 20 mL of fermentation broth was filtered with a piece of dried filter paper, followed by repeated washing for 3 times with deionized water. And then the wet biomass was transferred to the electric oven at 80 ℃ for 24 h until the constant weight was obtained, after that the dried biomass was weighed immediately.

### Reducing sugar assay

1 mL of the diluted fermentation supernatant was put in a 10 mL colorimetric tube, then 1.5 mL of 3,5-dinitrosalicylic acid (DNS) reagent was added. After mixing well, all the colorimetric tubes were put in boiling water bath for 5 min. When cooled to room temperature, all the reaction systems were diluted to 10 mL. After mixing well, the absorbance of each sample was measured at 550 nm. Glucose of different concentrations were prepared and measured by same method. And a glucose standard curve was acquired. Reducing sugar content (g/L) in the fermentation supernatant was calculated according to formula (2):

2 $${\text{Reducing sugar content }} = \left( {1.3941*\left( {A_{1} - A_{0} } \right) - 0.018} \right)*n,$$

where *A*_*1*_ is the absorbance of the sample, *A*_*0*_ is the absorbance of the corresponding control group, *n* is the dilution factor of the fermentation supernatant.

### Amino nitrogen assay

1 mL of fermentation supernatant was pipetted into a 150-mL conical flask. First, 30 mL of distilled water and 2 drops of methyl red indicator were added and adjusted to light red with 0.1 M hydrochloric acid, and left for 3 min. Next, adjusted to light yellow with 0.05 M sodium hydroxide solution. And then 5 mL of 18% neutral formaldehyde was added, mixed well and left for 3–5 min. Last, 4 drops of phenolphthalein indicator were added and titrated with 0.05 M sodium hydroxide solution to reddish color (pH 8.0–10.0). The amino nitrogen content (g/L) in the fermentation supernatant was calculated according to formula (3):

3 $${\text{Amino nitrogen }} = { }\frac{{c \times V_{1} \times 14}}{{V_{2} }} ,$$

where *c* is the concentration of NaOH solution (mol/L), *V*_*1*_ is the volume of NaOH solution (mL) used for titration, *V*_*2*_ is the volume of sample supernatant (mL) used for determination.

### Broth viscosity assay

About 15 mL of homogeneous fermentation broth was taken out, and then the viscosity was measured by LVDV-II + P viscometer with SC4-34 type rotor. And the specific parameters were as follows: the rotation speed was 200 rpm, and the temperature was room temperature.

### Determination of extracellular amino acids

The S-433D amino acid analyzer (Jiesheng Yike Technology Development Co., Ltd, Beijing, China) was used to determine the extracellular amino acids concentrations in the fermentation supernatant. A total of 800 μL of the fermentation supernatant was pipetted into a 1.5-mL EP tube, and then 200 μL of 10% sulfosalicylic acid was added. After mixing well, all the EP tubes were transferred at 4 °C for more than 30 min, then they were centrifuged at 13,000 rpm for 10 min. Next, the supernatant was diluted 3 times with lithium salt diluent, filtered through a 0.22-μm filter membrane, and poured into a liquid phase vial for testing. After that, 100 μL of the mixed amino acid standard and 900 μL of the lithium salt diluent were mixed well, and poured into the liquid phase vial for testing. In addition, 1000 μL lithium salt diluent was prepared as a blank. Finally, the free amino acid analysis standard program was called, the mixed amino acid standard was measured twice, then the lithium salt diluent was measured once, last the samples were measured.

### Determination of the pellet number and projected areas

The quantitative analysis method of *Aspergillus niger* established by Tang et al. (2015) was referenced in this study. To fix pellets sample, 1 mL of fermentation broth was mixed with 1 mL of fixing agent consisting of 40% formaldehyde and 60% ethanol. After mixing, 300 μL of the mixture was pipetted into a 5-mL centrifuge tube, deionized water was added and mixed, then the centrifuge tube was kept stand for 5 min. Next, the upper suspended hyphae were wiped off, the bottom pellets were washed three times repeatedly with deionized water and diluted to 5 mL finally. Afterwards, the pellets sample was poured into a disposable plate of 8.5 cm and mixed with 20 mL of 60 °C 0.05% agarose. After cooling, each sample was photographed with a camera under black background. Last, Image J software was used to process the photos for parameter determination.

## Results and discussion

### Selection of the optimal surfactant and its concentration

**Fig. 1:**
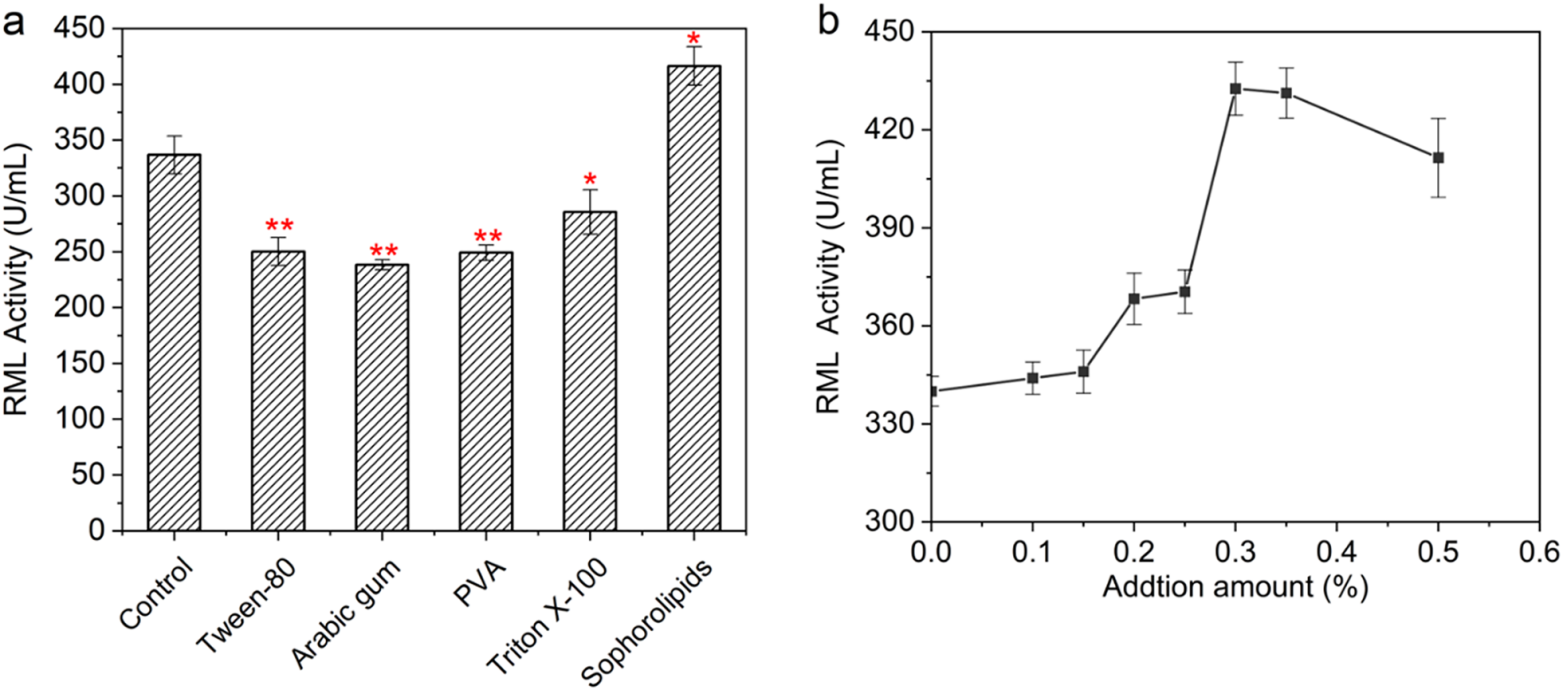
Selection of the optimal surfactant and its concentration. **a** Effect of different surfactants on RML activity (*: 0.01 < P ≤ 0.05; **: P ≤ 0.01). **b** Effect of different SLs concentrations on RML activity

Five surfactants, including Tween-80, acacia, polyvinyl alcohol, Triton X-100 and SLs, were tested in RML fermentation by *A. oryzae*. As shown in Fig. 1a, Tween-80, acacia, polyvinyl alcohol and Triton X-100 have strong inhibitory effect on the lipase production. The lowest enzyme activity dropped to about 70.0% of the control group by addition of acacia. In comparison, the addition of 0.2% SLs could significantly elevate the enzyme activity by 23.7%. Zhou et al. (2012) reported that acacia and polyvinyl alcohol could promote *A. oryzae* CJLU-31 to produce lipase. Liu et al. (2012) found that both Triton X-100 and acacia could promote *A. oryzae* WZ007 to produce lipase, and 0.2% acacia exhibited the best effect. Differently, herein acacia, polyvinyl alcohol and Triton X-100 could not increase the RML activity, which might be due to that the mechanism of surfactants on the enzyme is closely relevant to the non-homologous structure of lipase, extracellular secretion, enzymatic properties and physiological metabolic characteristics of the host. Therefore, the same surfactant has different impact on different hosts and lipases.

Subsequently, the addition amount of SLs was further optimized. It could be found that when SLs concentration was in the range of 0.1%–0.5%, it would not restrain *A. oryzae* from producing RML (Fig. 1b). When the addition amount of SLs was in the range of 0.1%–0.15%, the increase in enzyme activity did not exceed 2.0% compared to the control group. Once the concentration increased to 0.3%, the enzyme activity was significantly increased by 25.0%, however, as the content of SLs further increased, the increase in enzyme activity showed a decreasing trend. Therefore, 0.3% addition of SLs was adopted in the following experiments.

### Effects of SLs on the intracellular and extracellular RML enzyme activities

**Fig. 2:**
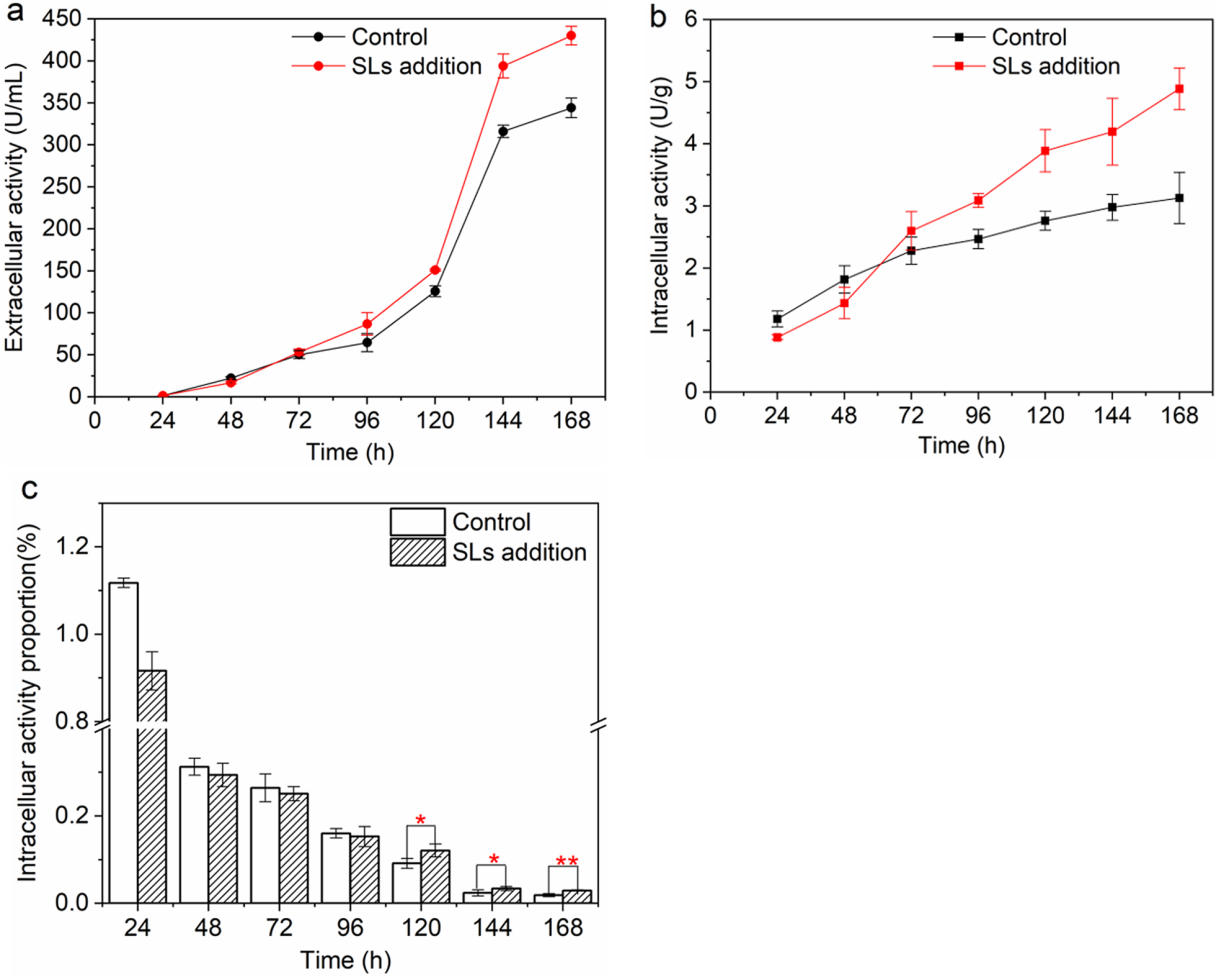
Comparison of RML activities between control group and SLs addition group. **a** Extracellular activity. **b** Intracellular activity. **c** Intracellular activity proportion (*: 0.01 < P ≤ 0.05; **: P ≤ 0.01) When the intracellular activity proportion was calculated, the units of intracellular and extracellular enzyme activity had been normalized into units in the whole fermentation broth

Although RML produced by *A. oryzae* is considered as an extracellular enzyme, it could also be detected intracellularly. The overall trends of the extracellular enzyme activity of both control group and experimental group (addition of 0.3% SLs) were described as follows. The extracellular enzyme activity increased over time during the whole fermentation process. When the cells entered the period of rapid enzyme production, especially after 120 h, the increase rate of extracellular enzyme activity between SLs addition group and the control group was significantly different. At the end of fermentation (168 h), the extracellular enzyme activity of SLs addition group was 430.0 U/mL, which was 25.0% higher than that of the control group (Fig. 2a). Although the intracellular enzyme activity accounted for a very low proportion of the total enzyme activity (sum of intracellular and extracellular enzyme activity), it also could reflect the overall enzyme production. Obviously, starting from 48 h, the increase rate of intracellular enzyme activity of SLs addition group exceeded that of the control group and lasted to the end of the fermentation (Fig. 2b). However, the highest intracellular enzyme activity accounted for only 1.1% of the total enzyme activity, indicating that the extracellular secretion of RML was relatively unhindered (Fig. 2c). Therefore, it was speculated that the addition of SLs would affect the cellular metabolism, and then promoted the RML synthesis, especially in the late stage (after 120 h). These results seemed to be different from previous reports that the amphiphilic structure of surfactants might change the permeability of cell membranes and promote the secretion of extracellular enzymes (Zhou 2012). In the following experiments, the effects of SLs on the viscosity of the fermentation broth and cell morphology during the RML fermentation process were further explored.

### Effects of SLs on the broth rheological properties, cell morphology and cellular physiological metabolism

**Fig. 3:**
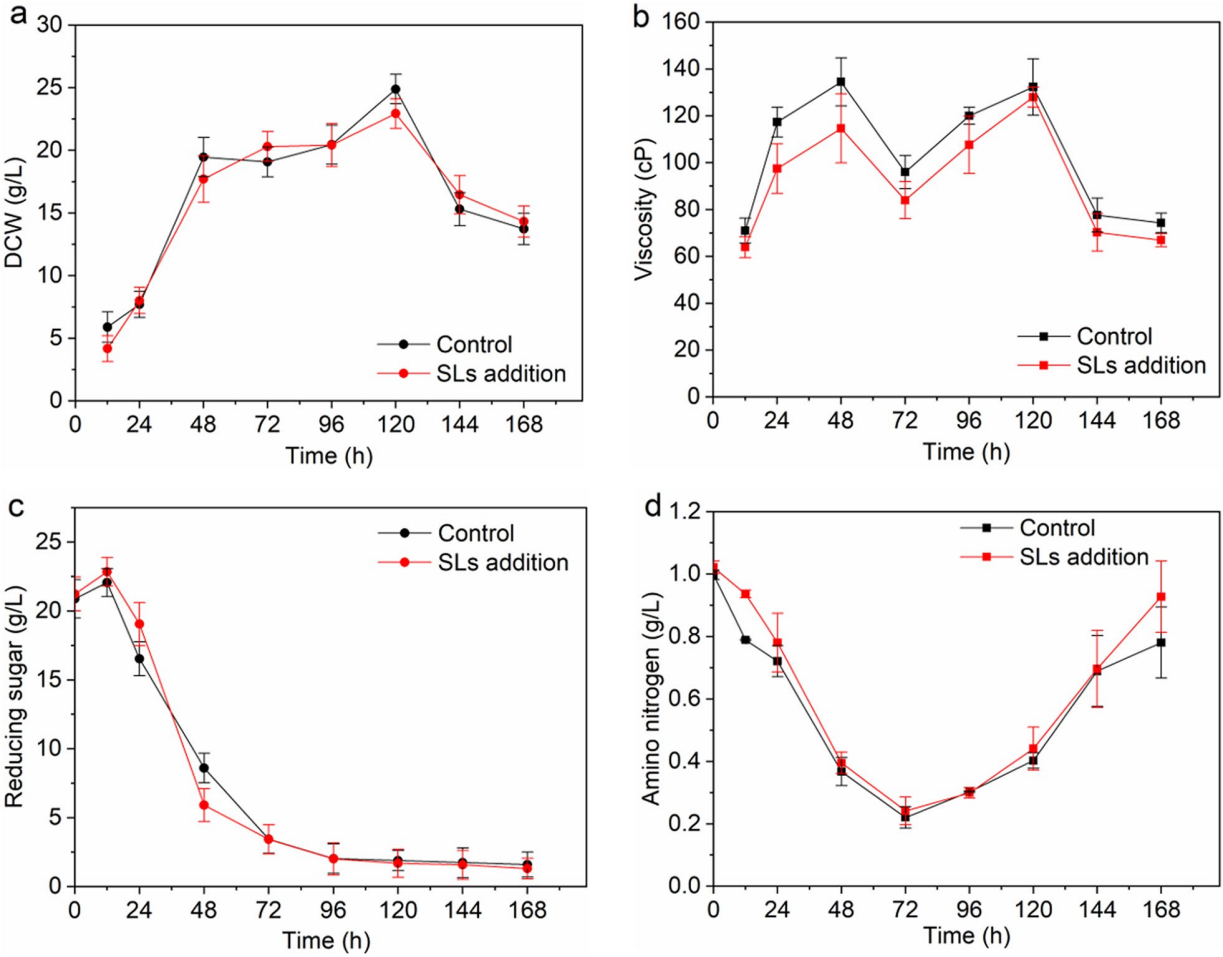
Profiles of basic parameters during RML fermentation between control group and SLs addition group. **a** DCW; **b** viscosity; **c** reducing sugar; **d** amino nitrogen

The overall trends of the biomass characterized by DCW of the control group and SLs addition group during the RML fermentation process were relatively consistent (Fig. 3a). At 12 h, DCW in SLs addition group was 29.0% lower than that in the control group, which indicated that SLs would inhibit the cell growth to a certain extent. The rapid growth period of *A. oryzae* in both groups was 24–48 h. Correspondingly, at this period, reducing sugar and amino nitrogen were consumed fast (Fig. 3c and d). DCW decreased after 120 h, which might be due to the autolysis of partially aged cells. During the fermentation process, the overall trend of the viscosity of the fermentation broth corresponded to the biomass (Fig. 3b). There was a phasic peak of viscosity at 48 h and 120 h, respectively. Different from the trend of DCW, the viscosity decreased rapidly at 48–72 h, which was mainly due to the significant change of morphology. Concretely, more dispersed hyphae aggregated into pellets, which greatly improved the rheological properties of the fermentation broth. It was worth noting that the viscosity of SLs addition group was lower than that of the control group during the whole fermentation process, especially during the early phase (24–72 h). This result indicated that SLs addition could reduce the viscosity of the fermentation broth, thereby improving the oxygen transfer in the environment. According to our previous work in a 50-L fermenter, the RML fermentation by *A. oryzae* is a highly aerobic process (data not shown), so starting from 48 h, the RML production rate of SLs addition group exceeded that of the control group and maintained higher level.

**Fig. 4:**
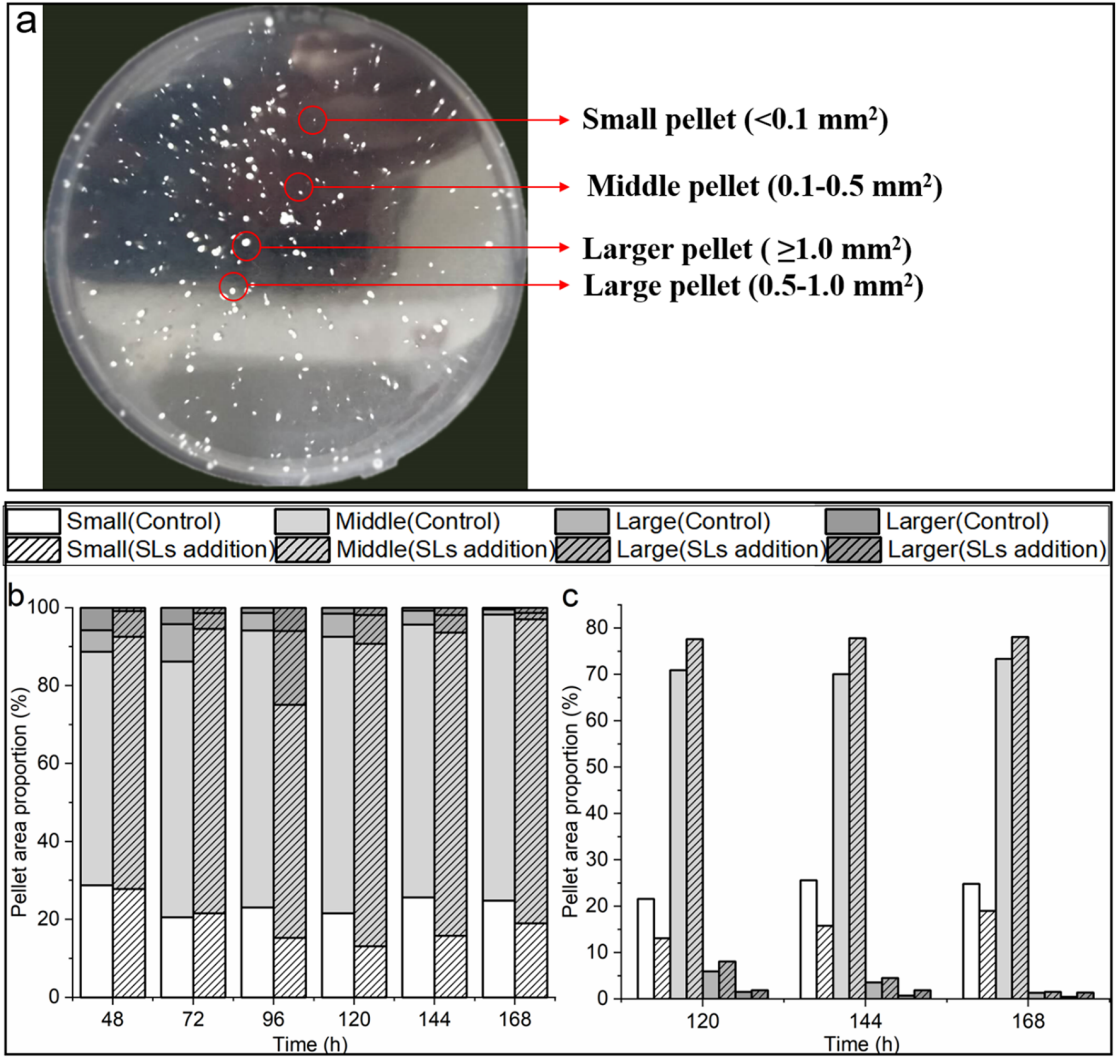
Definition of four types of pellets and comparison between control group and SLs addition group. **a** The photo of *A. oryzae* pellets laid on an agarose plate. **b** Stacked column chart showing the proportion and their change of four kinds of pellets during the whole fermentation process. **c** The bar graph showing the difference of four kinds of pellets proportion between the control group and SLs addition group during the rapid enzyme production period

The viscosity of the fermentation broth is not only related to biomass, but also closely related to morphology, and morphology is regulated by the cellular physiological metabolism and environmental parameters (Grimm et al. 2005; Riley et al. 2000). During RML fermentation process, *A. oryzae* exhibited different morphologies, ranging from dispersed mycelia to dense pellets, and simultaneously the sizes of pellets were different. Four types of pellets were defined according to the projection areas of pellets in this study (Fig. 4a). Small, middle, large and larger pellets refer to their projected areas < 0.1, 0.1–0.5, 0.5–1.0 and ≥ 1.0 mm^2^, respectively. Both in the control and SLs addition groups, middle pellets accounted for the vast majority, followed by the small pellets during the whole fermentation process (Fig. 4b). In terms of small pellets, the number exhibited a trend of first decreasing and then increasing, which might be due to the gradual enlargement of small pellets in the first and middle stages of fermentation. However, when the fermentation reached 120 h, the carbon source became insufficient, and the cells in the core parts of the large and larger pellets were aged, and then autolysis occurred which could provide nutrition and space for the growth of small pellets (White et al. 2002).

On the other hand, it was worth noting that compared to the control group, the proportion of small pellets was lower in SLs addition group, while the proportion of middle pellets was higher (Fig. 4c). These results indicated that the autolysis of the aging cells in SLs addition group provided more nutrients for small pellets to become middle pellets faster. And middle pellets were generally considered as an energetic and stable state for enzyme production. This might explain that the enzyme activity of SLs addition group was significantly higher than that of the control group during the period of rapid enzyme production. Chen et al. (2019) reported that the higher titer of L-malate by *A. oryzae* was achieved at a pellet number of 208/ml and a pellet diameter of 1.05 mm, not 1.12 mm or 1.02 mm, indicating that middle pellets might have larger pellet surface for transport and nutrient uptake, and smoother surface for reducing viscosity. In addition, the cell morphology affects cellular physiological metabolism, and amino acids are necessary precursors for RML synthesis, so cellular amino acids metabolism with the SLs addition was analyzed subsequently.

**Fig. 5:**
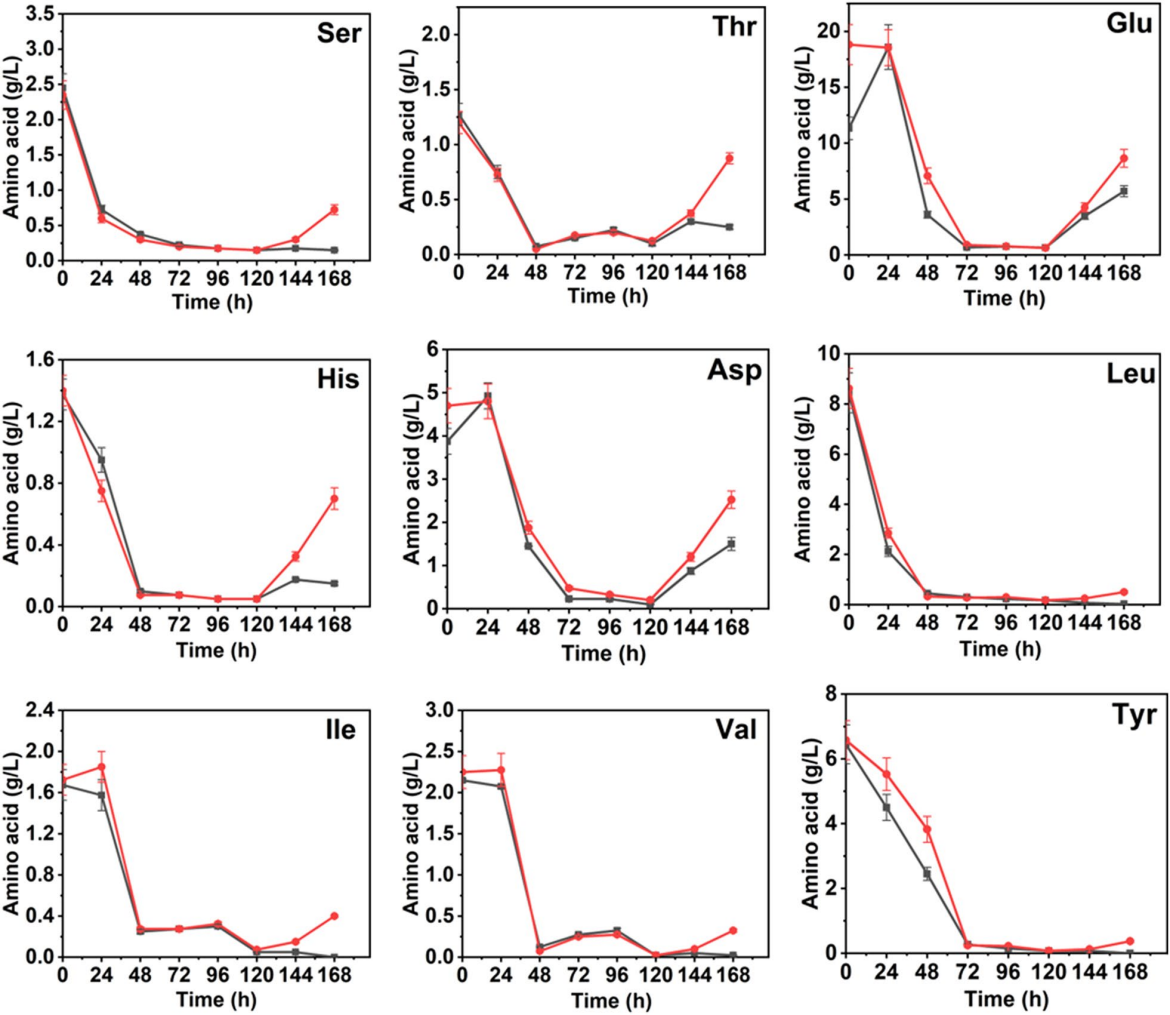
Profiles of extracellular amino acids during RML fermentation between control group and SLs addition group. Nine representative amino acids including serine, threonine, glutamate, aspartate, histidine, leucine, isoleucine, valine and tyrosine were shown. The control groups were represented by black lines, and the SLs addition groups were represented by red lines

The overall change trends of several extracellular amino acids concentrations were relatively consistent (Fig. 5). They were reduced to the minimum at 48 h, indicating that the cells in rapid growth phase consumed a large amount of amino acids in the medium to synthesize mycelia protein and related enzymes. The rebound phenomenon began to appear at 120 h, which may be due to the autolysis of aging cells. At the moment, protease activity increased, and more amino acids were released extracellularly, which was consistent with the rapid increase of the amino nitrogen content in the fermentation supernatant (Fig. 3d). Notably, after 120 h, most extracellular amino acids concentrations of SLs addition group were significantly higher than those of the control group, and the increase rates were also faster, especially serine, threonine, glutamate, aspartate and histidine. Among them, serine and threonine rank the first and second, respectively, in the amino acid composition of RML, while serine, histidine and aspartate are the components of the catalytic triad of RML. This phenomenon indicated that SLs addition group owned more precursor amino acids for RML synthesis. And this could also explain the RML enzyme activity and enzyme production rate of SLs addition group were significantly higher than those of the control group during the fast enzyme production period. Overall, SLs addition promoted the anabolism of RML precursor amino acids to a certain extent.

## Conclusions

Exogenous addition of 0.3% (w/v) SLs could significantly enhance the RML production by *A. oryzae*. In detail, with the addition of SLs, the viscosity of fermentation broth during the fermentation process was significantly reduced, and the proportion of middle pellets, which was considered as energetic and stable state, was increased, thereby improving the RML production. Otherwise, an increase in precursor amino acids synthesis during the fast enzyme production period also contributed to the improved RML activity with the addition of SLs. This work was believed to be helpful to extend this strategy for further industrial application.

## Data Availability

The data supporting the conclusions of this article are included in the main manuscript.

## Abbreviations

RML: *Rhizomucor miehei* Lipase
*A. oryzae*: *Aspergillus oryzae*
*p*-NP: *p*-Nitrophenol
*p*-NPP: *p*-Nitrophenol palmitate
PBS: Phosphate buffer saline
EDTA: Ethylene diamine tetraacetic acid
DCW: Dry cell weight
SLs: Sophorolipids
Ser: Serine
Thr: Threonine
Glu: Glutamate
His: Histidine
Asp: Aspartate
Leu: Leucine
Ile: Isoleucine
Val: Valine
Tyr: Tyrosine
